# OPENING MINDS THROUGH ART: CREATING UNIVERSITY PARTNERSHIPS THROUGH AN ADAPTIVE INTERGENERATIONAL ART PROGRAM

**DOI:** 10.1093/geroni/igae098.1505

**Published:** 2024-12-31

**Authors:** Amy Elliot, Katherine Abbott

**Affiliations:** Chair; Discussant

## Abstract

Opening Minds through Art (OMA) is an award-winning, evidence-based, intergenerational art-making program for people living with dementia. Developed in 2007 at Miami University’s Scripps Gerontology Center, the program is grounded in person-centered care principles and serves the vital need for people living with dementia to engage in creative expression and social connection. The current impact of OMA includes engagement through 1,100 trained facilitators in over 300 communities spanning 37 states and internationally. As a university-based program, OMA has maintained a focus on research with thirteen peer-reviewed studies to support the program’s impact on younger and older participants in areas such as more empathetic attitudes for other generations, improved mood, and increased engagement. Core to OMA’s success are relationships with 30 universities in the U.S. and globally. Through these partnerships, students engage with OMA through academic and service-learning opportunities to fortify their understanding of the aging process and empathy for older generations. Universities also benefit through community support and participation. The success of these programs is highly dependent on OMA’s ability to adapt to each university’s resources, programmatic interests, and community partnerships. This symposium will describe variations of OMA programming at three universities including work with health trainees at Penn State University, a student internship program at Florida State University, and the first and longest running OMA program including an innovative virtual variation at Miami University. Katy Abbott will lead a discussion of implementation, internal evaluation, and research components across programs and will explore future implications for university-based growth and adaptations.

